# Supplementary material for: The association of cardiovascular comorbidities with malignant and benign colorectal neoplasms
Source: PLoS One. 2026 Oct 21;21(10):e0356302. doi: 10.1371/journal.pone.0356302 (PMC13686501; doi:10.1371/journal.pone.0356302)
Supplement: Supplementary file 1 [file pone.0356302.s002.docx]

June 24, 2026

Re: “The Association of Cardiovascular Comorbidities with Malignant and Benign Colorectal Neoplasms”

Dear Reviewers,

Please find enclosed our revised manuscript to *PLOS One.*

We would like to thank the reviewers for their thoughtful comments and constructive feedback regarding this manuscript. We have thoroughly addressed all the reviewers’ comments and concerns; detailed responses and edits are provided below:

**Reviewer #1:** The study addresses an interesting and clinically relevant question using a large national dataset. However, several important methodological and interpretive concerns should be addressed before the manuscript can be considered for publication.

Major Comments

1. The manuscript repeatedly suggests that cardiovascular comorbidities may lead to earlier detection of colorectal cancer or more benign disease phenotypes, potentially influencing CRC screening strategies. However, the study design is cross-sectional and limited to inpatient admissions for lower GI bleeding. The NIS database does not allow assessment of: True cancer incidence, Time to diagnosis, Prior screening history, Stage at detection, and Medication exposure. Therefore, conclusions regarding earlier detection, stage migration, or implications for screening guidelines are not supported by the available data. The discussion and conclusion should be revised to reflect association rather than causation or detection mechanisms.

We appreciate this important point. We have revised the manuscript throughout to emphasize that our findings are associations only. We removed language implying causation, and we now explicitly state that the NIS data do not permit inference about time to diagnosis, true incidence, or stage migration (see lines 106-107; 234-237; 250-253; 268-269; 284-285).

2. A central biological explanation proposed in the manuscript is that patients with cardiovascular comorbidities are likely on antiplatelet or anticoagulation therapy, leading to increased bleeding and earlier lesion detection. However, outpatient medication data are not available in NIS. Cardiovascular comorbidities are therefore being used as a proxy for anticoagulant exposure, which may not be accurate. This limitation should be emphasized earlier and more clearly. The discussion should avoid mechanistic explanations that cannot be directly supported by the dataset.

Thank you for pointing this out. We agree that medication exposure cannot be ascertained with NIS data. We have added a clear statement in the Introduction that outpatient medication use (including antiplatelet/anticoagulant therapy) is not available and that cardiovascular comorbidities were used only as a proxy for possible higher likelihood of anticoagulant exposure. We removed speculative mechanistic language in the main text and reframed discussion of anticoagulation as a hypothesis requiring direct study (lines 106-110; 234-236).

3. Which covariates were included in the logistic regression models? Given the observational design, robust multivariable adjustment is essential. If models are unadjusted or minimally adjusted, this represents a major limitation and should be explicitly stated.

Explanation: Multivariable logistic regression included age, sex, and cardiovascular comorbidity status. Additional demographic and socioeconomic variables available in the NIS dataset (race, insurance status, income quartile, and geographic region) were not included in the original models. We acknowledge that residual confounding may therefore remain and have added this as a limitation in the Discussion and clarified the covariates in the Methods section.

Under *Methods*: “Logistic regression analyses were performed using age, sex, cardiovascular comorbidity status as independent variables. Additional demographics and socioeconomic characteristics available in the NIS database, including race/ethnicity, geographic region, income quartile, and insurance status, were not incorporated in the regression models.”

Under *Limitation:* “Furthermore, the regression analyses were only minimally adjusted, residual confounding cannot be excluded. Important demographic and socioeconomic factors such as race, insurance status, income level, and geographic region were not included in the regression models. Consequently, the observed associations should be interpreted as exploratory and hypothesis-generating.”

4. The Methods section states that multinomial logistic regression was used to evaluate associations with CRC stage. However, no stage-related results are presented in the Results section.

Thank you for pointing this out. CRC stage analyses were not included in the final study, and no stage-specific results were generated. Because we cannot retroactively gather a new data set that includes staging information, logistic regression was used to evaluate associations with colorectal cancer. This does represent a limitation of the study; however, we are planning to do a follow up study utilizing other databases that include this data specifically.

5. It should be emphasized more clearly that this study includes only patients admitted for lower GI bleeding. The findings, therefore, cannot be generalized

Thank you very much for pointing this out. We agree and have stated this explicitly during our limitation’s discussion (lines 294-297): “Furthermore, because NIS data is based on billing and administrative data for hospitalized patients, ICD-9 codes may not always accurately reflect clinical diagnoses or disease severity regarding CRC diagnoses and are specific to hospitalized patients. Therefore, the information may not be generalizable or applicable to the outpatient setting.”

**Reviewer #2:** This study addresses an interesting clinical question regarding the association between cardiovascular comorbidities and the diagnosis of colorectal neoplasms in patients presenting with lower gastrointestinal bleeding. However, several limitations reduce its current impact and applicability.

1. First, the dataset used (2007–2011) is relatively outdated. Given the substantial advancements in colorectal cancer screening, endoscopic techniques, and the evolving use of antiplatelet and anticoagulation therapies, it is difficult to determine how well these findings translate to current clinical practice.

Thank you for pointing this out. We agree that the current dataset evaluates early associations between CRC and cardiovascular comorbidities, and that guidelines and colonoscopy techniques have evolved in the following years. This is why we are pursuing a follow-up study that utilizes more recent data, as well as includes direct medication use, and CRC staging information.

2. Second, the research question is largely descriptive. While the study compares the likelihood of detecting benign versus malignant colorectal neoplasms based on cardiovascular comorbidity status, it does not sufficiently address clinically actionable implications. For example, reframing the question to evaluate how much more likely malignant colorectal neoplasms are to be detected in specific patient populations could provide more meaningful insights for risk stratification and clinical decision-making.

Thank you for your feedback. We absolutely agree, this is a descriptive study. Our goal with this study was to evaluate benign versus malignant CRC in association with cardiovascular comorbidities, to further evaluate the pre-test probability of CRC in patients taking anticoagulation and anti-platelet therapy, who may be more prone to bleeding, and therefore more prone to early detection. To further address more clinically applicable implications, we have included further insights regarding the study of aspirin in CRC, an important topic of discussion in current literature that dovetails well with our study. This further discussion can be found in lines (299-311):

“Furthermore, the regression analyses were only minimally adjusted, residual confounding cannot be excluded. Important demographic and socioeconomic factors such as race, insurance status, income level, and geographic region were not included in the regression models. Consequently, the observed associations should be interpreted as exploratory and hypothesis-generating. Due to the limitations of this cross-sectional study design, prospective studies and randomized controlled trials assessing CRC screening intervals in patients with or without cardiovascular comorbidities may provide more insight into early detection and outcomes related to CRC. Additionally, further research to stratify CRC diagnoses by stage at presentation can further validate the early detection hypothesis due to lower GI bleeding proposed in this paper.”

3. Lastly, the practical utility of the findings is limited, as the observed differences are relatively subtle. Methodologically, the study relies primarily on statistical associations without deeper analysis of confounders or mechanisms and lacks incorporation of additional relevant variables. The manuscript also would benefit from stronger engagement with existing literature. Overall, the work may be better suited as a descriptive report unless the authors refine the research question and apply more rigorous analytical approaches.

Thank you very much for your thoughts and feedback. We agree, and have therefore engaged further with current literature, as noted above (lines 312-324):

“The conclusions presented are based solely on the presence or absence of cardiovascular comorbidities. Medication-centered studies assessing the use of specific antiplatelet and anticoagulant regimes are also needed. Several studies have been done regarding the use of aspirin in CRC^23^, which dovetails well with this study. Specifically, in a large 2016 prospective cohort study of patients taking aspirin, the incidence of CRC was significantly lower compared to patients who were not taking aspirin. Although this study did not propose a mechanism, it did lead to several follow-up studies. More recent studies suggest aspirin use may be beneficial in CRC with specific tumor markers^24^, such as PI3K mutated tumors, while other studies suggest that all CRCs benefit from aspirin by mechanism of reduced inflammation^25^. While currently literature is still actively evaluating the efficacy and benefits of aspirin in CRC, this study encourages a broader look at CRC’s association with other anti-platelet and anticoagulant therapies and also contributes another proposed mechanism by which anticoagulation and anti-platelet therapy may contribute to a reduction in CRC rates.”

**Reviewer #3:**

4. The article is well written, the data is exhaustive and interesting, and the results are clinically relevant and have clinical application. Another reason for the observations could be that aspirin may reduce the risk of GI malignancy. This concept has existed for many years, though a cause-and-effect relationship could not be established. For example,
   Cao Y, Nishihara R, Wu K, Wang M, Ogino S, Willett WC, Spiegelman D, Fuchs CS, Giovannucci EL, Chan AT. Population-wide Impact of Long-term Use of Aspirin and the Risk for Cancer. JAMA Oncol. 2016 Jun 1;2(6):762-9. doi: 10.1001/jamaoncol.2015.6396. Erratum in: JAMA Oncol. 2019 Apr 1;5(4):579. doi: 10.1001/jamaoncol.2019.0286.
   This concept can be added to the discussion.
   Another method could be aspirin or anticoagulation-induced bleeding associated with early detection.

Thank you so much for this excellent addition, we have included further discussion of this and other relevant studies, and their implications for our own research (lines 312-324) and references 23-25.

Thank you for kindly reconsidering our revised manuscript for publication. We are sincerely grateful for the opportunity to resubmit and believe the revisions have significantly improved the manuscript in response to the reviewers’ insightful comments and suggestions. We hope we have adequately addressed all concerns would be pleased to make any additional revisions if needed.

Sincerely,

Farah Wani et al.
