# Supplementary material for: The association of cardiovascular comorbidities with malignant and benign colorectal neoplasms
Source: PLoS One. 2026 Oct 21;21(10):e0356302. doi: 10.1371/journal.pone.0356302 (PMC13686501; doi:10.1371/journal.pone.0356302)
Supplement: Supplementary file 2 [file pone.0356302.s003.docx]

August 29, 2026

Re: “The Association of Cardiovascular Comorbidities with Malignant and Benign Colorectal Neoplasms”

Dear Reviewers,

Please find enclosed our revised manuscript to *PLOS One.*

We would like to thank the reviewers for their thoughtful comments and constructive feedback regarding this manuscript. We have thoroughly addressed all the reviewers’ comments and concerns; detailed responses and edits are provided below:

**Reviewer #2:** The authors have adequately addressed all of my concerns. I have one suggestion: currently, all of the data are presented in tables, which makes the manuscript somewhat exhausting to read. Presenting some of the key data in figure format would improve readability and help readers more easily interpret the findings.

**Response:** We thank the reviewers for this helpful suggestion. We agree that presenting key findings visually improves the readability and facilitates efficient interpretation of the results. In response, we have added new figures (Figure 1 and 2) and combined Tables 2-6 into Tables 2 and 3. The Results and Discussion section have been revised to reference the new figures and tables where appropriate.

We thank the reviewers and editing team for reconsidering and giving us valuable feedback for this manuscript. We are sincerely appreciative for the opportunity to resubmit and believe the revisions have significantly improved the manuscript in response to the reviewers’ comments. WE hope that our revisions have adequately addressed all concerns, and we welcome any additional feedback or requested revisions.

Sincerely,

Farah Wani et al.
