# Supplementary material for: The association of cardiovascular comorbidities with malignant and benign colorectal neoplasms
Source: PLoS One. 2026 Oct 21;21(10):e0356302. doi: 10.1371/journal.pone.0356302 (PMC13686501; doi:10.1371/journal.pone.0356302)
Supplement: Peer review history

# Peer review: Decision Letter 0

**DOI:** 10.1371/journal.pone.0356302.r001

**Authors:** Yongzhong Guo

3 May 2026

Dear Dr. Marrufo,

Thank you for submitting your manuscript to PLOS ONE. After careful consideration, we feel that it has merit but does not fully meet PLOS ONE’s publication criteria as it currently stands. Therefore, we invite you to submit a revised version of the manuscript that addresses the points raised during the review process.

Please submit your revised manuscript by Jun 17 2026 11:59PM. If you will need more time than this to complete your revisions, please reply to this message or contact the journal office at plosone@plos.org. When you're ready to submit your revision, log on to https://www.editorialmanager.com/pone/ and select the 'Submissions Needing Revision' folder to locate your manuscript file.

- A letter that responds to each point raised by the academic editor and reviewer(s). You should upload this letter as a separate file labeled 'Response to Reviewers'.
- A marked-up copy of your manuscript that highlights changes made to the original version. You should upload this as a separate file labeled 'Revised Manuscript with Track Changes'.
- An unmarked version of your revised paper without tracked changes. You should upload this as a separate file labeled 'Manuscript'.

If you would like to make changes to your financial disclosure, please include your updated statement in your cover letter. Guidelines for resubmitting your figure files are available below the reviewer comments at the end of this letter.

If applicable, we recommend that you deposit your laboratory protocols in protocols.io to enhance the reproducibility of your results. Protocols.io assigns your protocol its own identifier (DOI) so that it can be cited independently in the future. For instructions see: https://journals.plos.org/plosone/s/submission-guidelines#loc-laboratory-protocols. Additionally, PLOS ONE offers an option for publishing peer-reviewed Lab Protocol articles, which describe protocols hosted on protocols.io. Read more information on sharing protocols at https://plos.org/protocols?utm_medium=editorial-email&utm_source=authorletters&utm_campaign=protocols.

As the corresponding author, your ORCID iD is verified in the submission system and will appear in the published article. PLOS supports the use of ORCID, and we encourage all coauthors to register for an ORCID iD and use it as well. Please encourage your coauthors to verify their ORCID iD within the submission system before final acceptance, as unverified ORCID iDs will not appear in the published article. *Only*  the individual author can complete the verification step; PLOS staff *cannot*  verify ORCID iDs on behalf of authors.

We look forward to receiving your revised manuscript.

Kind regards,

Yongzhong Guo, Ph.D

Academic Editor

PLOS One

Journal Requirements:

When submitting your revision, we need you to address these additional requirements.

1. Please ensure that your manuscript meets PLOS ONE's style requirements, including those for file naming. The PLOS ONE style templates can be found at

https://journals.plos.org/plosone/s/file?id=wjVg/PLOSOne_formatting_sample_main_body.pdf and

https://journals.plos.org/plosone/s/file?id=ba62/PLOSOne_formatting_sample_title_authors_affiliations.pdf

2. Please note that your Data Availability Statement is currently missing a direct link to access each database. If your manuscript is accepted for publication, you will be asked to provide these details on a very short timeline. We therefore suggest that you provide this information now, though we will not hold up the peer review process if you are unable.

3. If the reviewer comments include a recommendation to cite specific previously published works, please review and evaluate these publications to determine whether they are relevant and should be cited. There is no requirement to cite these works unless the editor has indicated otherwise.

[Note: HTML markup is below. Please do not edit.]

Reviewers' comments:

Reviewer's Responses to Questions

**Comments to the Author**

1. Is the manuscript technically sound, and do the data support the conclusions?

Reviewer #1: Yes

Reviewer #2: Partly

Reviewer #3: Yes

**********

2. Has the statistical analysis been performed appropriately and rigorously?

Reviewer #1: Yes

Reviewer #2: N/A

Reviewer #3: Yes

**********

3. Have the authors made all data underlying the findings in their manuscript fully available?

The PLOS Data policy

Reviewer #1: Yes

Reviewer #2: Yes

Reviewer #3: Yes

**********

4. Is the manuscript presented in an intelligible fashion and written in standard English?

Reviewer #1: Yes

Reviewer #2: Yes

Reviewer #3: Yes

**********

Reviewer #1: The study addresses an interesting and clinically relevant question using a large national dataset. However, several important methodological and interpretive concerns should be addressed before the manuscript can be considered for publication.

Major Comments

1. The manuscript repeatedly suggests that cardiovascular comorbidities may lead to earlier detection of colorectal cancer or more benign disease phenotypes, potentially influencing CRC screening strategies. However, the study design is cross-sectional and limited to inpatient admissions for lower GI bleeding. The NIS database does not allow assessment of: True cancer incidence, Time to diagnosis, Prior screening history, Stage at detection, and Medication exposure. Therefore, conclusions regarding earlier detection, stage migration, or implications for screening guidelines are not supported by the available data. The discussion and conclusion should be revised to reflect association rather than causation or detection mechanisms.

2. A central biological explanation proposed in the manuscript is that patients with cardiovascular comorbidities are likely on antiplatelet or anticoagulation therapy, leading to increased bleeding and earlier lesion detection. However, outpatient medication data are not available in NIS. Cardiovascular comorbidities are therefore being used as a proxy for anticoagulant exposure, which may not be accurate. This limitation should be emphasised earlier and more clearly. The discussion should avoid mechanistic explanations that cannot be directly supported by the dataset.

3. Which covariates were included in the logistic regression models? Given the observational design, robust multivariable adjustment is essential. If models are unadjusted or minimally adjusted, this represents a major limitation and should be explicitly stated.

4. The Methods section states that multinomial logistic regression was used to evaluate associations with CRC stage. However, no stage-related results are presented in the Results section.

5. It should be emphasised more clearly that this study includes only patients admitted for lower GI bleeding. The findings, therefore, cannot be generalised

Reviewer #2: This study addresses an interesting clinical question regarding the association between cardiovascular comorbidities and the diagnosis of colorectal neoplasms in patients presenting with lower gastrointestinal bleeding. However, several limitations reduce its current impact and applicability.

First, the dataset used (2007–2011) is relatively outdated. Given the substantial advancements in colorectal cancer screening, endoscopic techniques, and the evolving use of antiplatelet and anticoagulation therapies, it is difficult to determine how well these findings translate to current clinical practice.

Second, the research question is largely descriptive. While the study compares the likelihood of detecting benign versus malignant colorectal neoplasms based on cardiovascular comorbidity status, it does not sufficiently address clinically actionable implications. For example, reframing the question to evaluate how much more likely malignant colorectal neoplasms are to be detected in specific patient populations could provide more meaningful insights for risk stratification and clinical decision-making.

Lastly, the practical utility of the findings is limited, as the observed differences are relatively subtle. Methodologically, the study relies primarily on statistical associations without deeper analysis of confounders or mechanisms, and lacks incorporation of additional relevant variables. The manuscript also would benefit from stronger engagement with existing literature.

Overall, the work may be better suited as a descriptive report unless the authors refine the research question and apply more rigorous analytical approaches.

Reviewer #3: The article is well written, the data is exhaustive and interesting, and the results are clinically relevant and have clinical application. Another reason for the observations could be that aspirin may reduce the risk of GI malignancy. This concept has existed for many years, though a cause-and-effect relationship could not be established. For example,

Cao Y, Nishihara R, Wu K, Wang M, Ogino S, Willett WC, Spiegelman D, Fuchs CS, Giovannucci EL, Chan AT. Population-wide Impact of Long-term Use of Aspirin and the Risk for Cancer. JAMA Oncol. 2016 Jun 1;2(6):762-9. doi: 10.1001/jamaoncol.2015.6396. Erratum in: JAMA Oncol. 2019 Apr 1;5(4):579. doi: 10.1001/jamaoncol.2019.0286.

This concept can be added to the discussion.

Another method could be aspirin or anticoagulation-induced bleeding associated with early detection.

**********

what does this mean?). If published, this will include your full peer review and any attached files.

If you choose “no”, your identity will remain anonymous but your review may still be made public.

**Do you want your identity to be public for this peer review?**  For information about this choice, including consent withdrawal, please see our Privacy Policy

Reviewer #1: No

Reviewer #2: No

Reviewer #3: **Yes:**  Mark Christopher Arokiaraj

**********

[NOTE: If reviewer comments were submitted as an attachment file, they will be attached to this email and accessible via the submission site. Please log into your account, locate the manuscript record, and check for the action link "View Attachments". If this link does not appear, there are no attachment files.]

To ensure your figures meet our technical requirements, please review our figure guidelines: https://journals.plos.org/plosone/s/figures

You may also use PLOS’s free figure tool, NAAS, to help you prepare publication quality figures: https://journals.plos.org/plosone/s/figures#loc-tools-for-figure-preparation.

NAAS will assess whether your figures meet our technical requirements by comparing each figure against our figure specifications.

# Author comment: Author response to Decision Letter 1

**DOI:** 10.1371/journal.pone.0356302.r002

2 Jul 2026

June 24, 2026

Re: “The Association of Cardiovascular Comorbidities with Malignant and Benign Colorectal Neoplasms”

Dear Reviewers,

Please find enclosed our revised manuscript to PLOS One.

We would like to thank the reviewers for their thoughtful comments and constructive feedback regarding this manuscript. We have thoroughly addressed all the reviewers’ comments and concerns; detailed responses and edits are provided below:

Reviewer #1: The study addresses an interesting and clinically relevant question using a large national dataset. However, several important methodological and interpretive concerns should be addressed before the manuscript can be considered for publication.

Major Comments

1. The manuscript repeatedly suggests that cardiovascular comorbidities may lead to earlier detection of colorectal cancer or more benign disease phenotypes, potentially influencing CRC screening strategies. However, the study design is cross-sectional and limited to inpatient admissions for lower GI bleeding. The NIS database does not allow assessment of: True cancer incidence, Time to diagnosis, Prior screening history, Stage at detection, and Medication exposure. Therefore, conclusions regarding earlier detection, stage migration, or implications for screening guidelines are not supported by the available data. The discussion and conclusion should be revised to reflect association rather than causation or detection mechanisms.

We appreciate this important point. We have revised the manuscript throughout to emphasize that our findings are associations only. We removed language implying causation, and we now explicitly state that the NIS data do not permit inference about time to diagnosis, true incidence, or stage migration (see lines 106-107; 234-237; 250-253; 268-269; 284-285).

2. A central biological explanation proposed in the manuscript is that patients with cardiovascular comorbidities are likely on antiplatelet or anticoagulation therapy, leading to increased bleeding and earlier lesion detection. However, outpatient medication data are not available in NIS. Cardiovascular comorbidities are therefore being used as a proxy for anticoagulant exposure, which may not be accurate. This limitation should be emphasized earlier and more clearly. The discussion should avoid mechanistic explanations that cannot be directly supported by the dataset.

Thank you for pointing this out. We agree that medication exposure cannot be ascertained with NIS data. We have added a clear statement in the Introduction that outpatient medication use (including antiplatelet/anticoagulant therapy) is not available and that cardiovascular comorbidities were used only as a proxy for possible higher likelihood of anticoagulant exposure. We removed speculative mechanistic language in the main text and reframed discussion of anticoagulation as a hypothesis requiring direct study (lines 106-110; 234-236).

3. Which covariates were included in the logistic regression models? Given the observational design, robust multivariable adjustment is essential. If models are unadjusted or minimally adjusted, this represents a major limitation and should be explicitly stated.

Explanation: Multivariable logistic regression included age, sex, and cardiovascular comorbidity status. Additional demographic and socioeconomic variables available in the NIS dataset (race, insurance status, income quartile, and geographic region) were not included in the original models. We acknowledge that residual confounding may therefore remain and have added this as a limitation in the Discussion and clarified the covariates in the Methods section.

Under Methods: “Logistic regression analyses were performed using age, sex, cardiovascular comorbidity status as independent variables. Additional demographics and socioeconomic characteristics available in the NIS database, including race/ethnicity, geographic region, income quartile, and insurance status, were not incorporated in the regression models.”

Under Limitation: “Furthermore, the regression analyses were only minimally adjusted, residual confounding cannot be excluded. Important demographic and socioeconomic factors such as race, insurance status, income level, and geographic region were not included in the regression models. Consequently, the observed associations should be interpreted as exploratory and hypothesis-generating.”

4. The Methods section states that multinomial logistic regression was used to evaluate associations with CRC stage. However, no stage-related results are presented in the Results section.

Thank you for pointing this out. CRC stage analyses were not included in the final study, and no stage-specific results were generated. Because we cannot retroactively gather a new data set that includes staging information, logistic regression was used to evaluate associations with colorectal cancer. This does represent a limitation of the study; however, we are planning to do a follow up study utilizing other databases that include this data specifically.

5. It should be emphasized more clearly that this study includes only patients admitted for lower GI bleeding. The findings, therefore, cannot be generalized

Thank you very much for pointing this out. We agree and have stated this explicitly during our limitation’s discussion (lines 294-297): “Furthermore, because NIS data is based on billing and administrative data for hospitalized patients, ICD-9 codes may not always accurately reflect clinical diagnoses or disease severity regarding CRC diagnoses and are specific to hospitalized patients. Therefore, the information may not be generalizable or applicable to the outpatient setting.”

Reviewer #2: This study addresses an interesting clinical question regarding the association between cardiovascular comorbidities and the diagnosis of colorectal neoplasms in patients presenting with lower gastrointestinal bleeding. However, several limitations reduce its current impact and applicability.

1. First, the dataset used (2007–2011) is relatively outdated. Given the substantial advancements in colorectal cancer screening, endoscopic techniques, and the evolving use of antiplatelet and anticoagulation therapies, it is difficult to determine how well these findings translate to current clinical practice.

Thank you for pointing this out. We agree that the current dataset evaluates early associations between CRC and cardiovascular comorbidities, and that guidelines and colonoscopy techniques have evolved in the following years. This is why we are pursuing a follow-up study that utilizes more recent data, as well as includes direct medication use, and CRC staging information.

2. Second, the research question is largely descriptive. While the study compares the likelihood of detecting benign versus malignant colorectal neoplasms based on cardiovascular comorbidity status, it does not sufficiently address clinically actionable implications. For example, reframing the question to evaluate how much more likely malignant colorectal neoplasms are to be detected in specific patient populations could provide more meaningful insights for risk stratification and clinical decision-making.

Thank you for your feedback. We absolutely agree, this is a descriptive study. Our goal with this study was to evaluate benign versus malignant CRC in association with cardiovascular comorbidities, to further evaluate the pre-test probability of CRC in patients taking anticoagulation and anti-platelet therapy, who may be more prone to bleeding, and therefore more prone to early detection. To further address more clinically applicable implications, we have included further insights regarding the study of aspirin in CRC, an important topic of discussion in current literature that dovetails well with our study. This further discussion can be found in lines (299-311):

“Furthermore, the regression analyses were only minimally adjusted, residual confounding cannot be excluded. Important demographic and socioeconomic factors such as race, insurance status, income level, and geographic region were not included in the regression models. Consequently, the observed associations should be interpreted as exploratory and hypothesis-generating. Due to the limitations of this cross-sectional study design, prospective studies and randomized controlled trials assessing CRC screening intervals in patients with or without cardiovascular comorbidities may provide more insight into early detection and outcomes related to CRC. Additionally, further research to stratify CRC diagnoses by stage at presentation can further validate the early detection hypothesis due to lower GI bleeding proposed in this paper.”

3. Lastly, the practical utility of the findings is limited, as the observed differences are relatively subtle. Methodologically, the study relies primarily on statistical associations without deeper analysis of confounders or mechanisms and lacks incorporation of additional relevant variables. The manuscript also would benefit from stronger engagement with existing literature. Overall, the work may be better suited as a descriptive report unless the authors refine the research question and apply more rigorous analytical approaches.

Thank you very much for your thoughts and feedback. We agree, and have therefore engaged further with current literature, as noted above (lines 312-324):

“The conclusions presented are based solely on the presence or absence of cardiovascular comorbidities. Medication-centered studies assessing the use of specific antiplatelet and anticoagulant regimes are also needed. Several studies have been done regarding the use of aspirin in CRC23, which dovetails well with this study. Specifically, in a large 2016 prospective cohort study of patients taking aspirin, the incidence of CRC was significantly lower compared to patients who were not taking aspirin. Although this study did not propose a mechanism, it did lead to several follow-up studies. More recent studies suggest aspirin use may be beneficial in CRC with specific tumor markers24, such as PI3K mutated tumors, while other studies suggest that all CRCs benefit from aspirin by mechanism of reduced inflammation25. While currently literature is still actively evaluating the efficacy and benefits of aspirin in CRC, this study encourages a broader look at CRC’s association with other anti-platelet and anticoagulant therapies and also contributes another proposed mechanism by which anticoagulation and anti-platelet therapy may contribute to a reduction in CRC rates.”

Reviewer #3:

4. The article is well written, the data is exhaustive and interesting, and the results are clinically relevant and have clinical application. Another reason for the observations could be that aspirin may reduce the risk of GI malignancy. This concept has existed for many years, though a cause-and-effect relationship could not be established. For example,

Cao Y, Nishihara R, Wu K, Wang M, Ogino S, Willett WC, Spiegelman D, Fuchs CS, Giovannucci EL, Chan AT. Population-wide Impact of Long-term Use of Aspirin and the Risk for Cancer. JAMA Oncol. 2016 Jun 1;2(6):762-9. doi: 10.1001/jamaoncol.2015.6396. Erratum in: JAMA Oncol. 2019 Apr 1;5(4):579. doi: 10.1001/jamaoncol.2019.0286.

This concept can be added to the discussion.

Another method could be aspirin or anticoagulation-induced bleeding associated with early detection.

Thank you so much for this excellent addition, we have included further discussion of this and other relevant studies, and their implications for our own research (lines 312-324) and references 23-25.

Thank you for kindly reconsidering our revised manuscript for publication. We are sincerely grateful for the opportunity to resubmit and believe the revisions have significantly improved the manuscript in response to the reviewers’ insightful comments and suggestions. We hope we have adequately addressed all concerns would be pleased to make any additional revisions if needed.

Sincerely,

Farah Wani et al.

Attachment Submitted filename: CRC_Reviewer Comment Responses.docx

# Peer review: Decision Letter 1

**DOI:** 10.1371/journal.pone.0356302.r003

**Authors:** Yongzhong Guo

26 Aug 2026

Dear Dr. Marrufo,

Thank you for submitting your manuscript to PLOS One. After careful consideration, we feel that it has merit but does not fully meet PLOS One’s publication criteria as it currently stands. Therefore, we invite you to submit a revised version of the manuscript that addresses the points raised during the review process.

Please submit your revised manuscript by Oct 10 2026 11:59PM. If you will need more time than this to complete your revisions, please reply to this message or contact the journal office at plosone@plos.org. When you're ready to submit your revision, log on to https://www.editorialmanager.com/pone/ and select the 'Submissions Needing Revision' folder to locate your manuscript file.

- A letter that responds to each point raised by the academic editor and reviewer(s). You should upload this letter as a separate file labeled 'Response to Reviewers'.
- A marked-up copy of your manuscript that highlights changes made to the original version. You should upload this as a separate file labeled 'Revised Manuscript with Track Changes'.
- An unmarked version of your revised paper without tracked changes. You should upload this as a separate file labeled 'Manuscript'.

If you would like to make changes to your financial disclosure, please include your updated statement in your cover letter. Guidelines for resubmitting your figure files are available below the reviewer comments at the end of this letter.

If applicable, we recommend that you deposit your laboratory protocols in protocols.io to enhance the reproducibility of your results. Protocols.io assigns your protocol its own identifier (DOI) so that it can be cited independently in the future. For instructions see: https://journals.plos.org/plosone/s/submission-guidelines#loc-laboratory-protocols. Additionally, PLOS One offers an option for publishing peer-reviewed Lab Protocol articles, which describe protocols hosted on protocols.io. Read more information on sharing protocols at https://plos.org/protocols?utm_medium=editorial-email&utm_source=authorletters&utm_campaign=protocols.

As the corresponding author, your ORCID iD is verified in the submission system and will appear in the published article. PLOS supports the use of ORCID, and we encourage all coauthors to register for an ORCID iD and use it as well. Please encourage your coauthors to verify their ORCID iD within the submission system before final acceptance, as unverified ORCID iDs will not appear in the published article. *Only*  the individual author can complete the verification step; PLOS staff *cannot*  verify ORCID iDs on behalf of authors.

We look forward to receiving your revised manuscript.

Kind regards,

Yongzhong Guo, Ph.D

Academic Editor

PLOS One

Journal Requirements:

If the reviewer comments include a recommendation to cite specific previously published works, please review and evaluate these publications to determine whether they are relevant and should be cited. There is no requirement to cite these works unless the editor has indicated otherwise.

Please review your reference list to ensure that it is complete and correct. If you have cited papers that have been retracted, please include the rationale for doing so in the manuscript text, or remove these references and replace them with relevant current references. Any changes to the reference list should be mentioned in the rebuttal letter that accompanies your revised manuscript. If you need to cite a retracted article, indicate the article’s retracted status in the References list and also include a citation and full reference for the retraction notice.

[Note: HTML markup is below. Please do not edit.]

Reviewers' comments:

Reviewer's Responses to Questions

**Comments to the Author**

Reviewer #2: All comments have been addressed

Reviewer #3: All comments have been addressed

**********

2. Is the manuscript technically sound, and do the data support the conclusions?

Reviewer #2: Partly

Reviewer #3: Yes

**********

3. Has the statistical analysis been performed appropriately and rigorously?

Reviewer #2: Yes

Reviewer #3: Yes

**********

4. Have the authors made all data underlying the findings in their manuscript fully available?

The PLOS Data policy

Reviewer #2: Yes

Reviewer #3: Yes

**********

5. Is the manuscript presented in an intelligible fashion and written in standard English?

Reviewer #2: Yes

Reviewer #3: Yes

**********

Reviewer #2: The authors have adequately addressed all of my concerns. I have one suggestion: currently, all of the data are presented in tables, which makes the manuscript somewhat exhausting to read. Presenting some of the key data in figure format would improve readability and help readers more easily interpret the findings.

Reviewer #3: The article is written well and all the necessary changes have been included. The observed associations can be verified by large studies in the future.

**********

what does this mean?). If published, this will include your full peer review and any attached files.

If you choose “no”, your identity will remain anonymous but your review may still be made public.

**Do you want your identity to be public for this peer review?**  For information about this choice, including consent withdrawal, please see our Privacy Policy

Reviewer #2: No

Reviewer #3: **Yes:**  Marki Christopher Arokiaraj

**********

[NOTE: If reviewer comments were submitted as an attachment file, they will be attached to this email and accessible via the submission site. Please log into your account, locate the manuscript record, and check for the action link "View Attachments". If this link does not appear, there are no attachment files.]

To ensure your figures meet our technical requirements, please review our figure guidelines: https://journals.plos.org/plosone/s/figures

You may also use PLOS’s free figure tool, NAAS, to help you prepare publication quality figures: https://journals.plos.org/plosone/s/figures#loc-tools-for-figure-preparation.

NAAS will assess whether your figures meet our technical requirements by comparing each figure against our figure specifications.

# Author comment: Author response to Decision Letter 2

**DOI:** 10.1371/journal.pone.0356302.r004

30 Aug 2026

August 29, 2026

Re: “The Association of Cardiovascular Comorbidities with Malignant and Benign Colorectal Neoplasms”

Dear Reviewers,

Please find enclosed our revised manuscript to PLOS One. We would like to thank the reviewers for their thoughtful comments and constructive feedback regarding this manuscript. We have thoroughly addressed all the reviewers’ comments and concerns; detailed responses and edits are provided below:

Reviewer #2: The authors have adequately addressed all of my concerns. I have one suggestion: currently, all of the data are presented in tables, which makes the manuscript somewhat exhausting to read. Presenting some of the key data in figure format would improve readability and help readers more easily interpret the findings.

Response: We thank the reviewers for this helpful suggestion. We agree that presenting key findings visually improves the readability and facilitates efficient interpretation of the results. In response, we have added new figures (Figure 1 and 2) and combined Tables 2-6 into Tables 2 and 3. The Results and Discussion section have been revised to reference the new figures and tables where appropriate.

We thank the reviewers and editing team for reconsidering and giving us valuable feedback for this manuscript. We are sincerely appreciative for the opportunity to resubmit and believe the revisions have significantly improved the manuscript in response to the reviewers’ comments. WE hope that our revisions have adequately addressed all concerns, and we welcome any additional feedback or requested revisions.

Sincerely,

Farah Wani et al.

Attachment Submitted filename: Colorectal Manuscript_Updated.docx

# Peer review: Decision Letter 2

**DOI:** 10.1371/journal.pone.0356302.r005

**Authors:** Yongzhong Guo

21 Sep 2026

The Association of Cardiovascular Comorbidities with Malignant and Benign Colorectal Neoplasms

PONE-D-25-69189R2

Dear Dr. Marrufo,

We’re pleased to inform you that your manuscript has been judged scientifically suitable for publication and will be formally accepted for publication once it meets all outstanding technical requirements.

Within one week, you’ll receive an e-mail detailing the required amendments. When these have been addressed, you’ll receive a formal acceptance letter and your manuscript will be scheduled for publication.

An invoice will be generated when your article is formally accepted. Please note, if your institution has a publishing partnership with PLOS and your article meets the relevant criteria, all or part of your publication costs will be covered. Please make sure your user information is up-to-date by logging into Editorial Manager at Editorial Manager® and clicking the ‘Update My Information' link at the top of the page. For questions related to billing, please contact billing support.

If your institution or institutions have a press office, please notify them about your upcoming paper to help maximize its impact. If they’ll be preparing press materials, please inform our press team as soon as possible -- no later than 48 hours after receiving the formal acceptance. Your manuscript will remain under strict press embargo until 2 pm Eastern Time on the date of publication. For more information, please contact onepress@plos.org.

Kind regards,

Yongzhong Guo, Ph.D

Academic Editor

PLOS One

Additional Editor Comments (optional):

Reviewers' comments:

Reviewer's Responses to Questions

**Comments to the Author**

Reviewer #2: All comments have been addressed

**********

2. Is the manuscript technically sound, and do the data support the conclusions?

Reviewer #2: Yes

**********

3. Has the statistical analysis been performed appropriately and rigorously?

Reviewer #2: Yes

**********

4. Have the authors made all data underlying the findings in their manuscript fully available?

The PLOS Data policy

Reviewer #2: No

**********

5. Is the manuscript presented in an intelligible fashion and written in standard English?

Reviewer #2: Yes

**********

Reviewer #2: In this second revision, the authors have adequately addressed the concerns raised in my previous review. Based on the revised manuscript and the authors’ responses, I have no further comments at this time.

**********

what does this mean?). If published, this will include your full peer review and any attached files.

If you choose “no”, your identity will remain anonymous but your review may still be made public.

**Do you want your identity to be public for this peer review?**  For information about this choice, including consent withdrawal, please see our Privacy Policy

Reviewer #2: No

**********

# Editor report: Acceptance letter

**DOI:** 10.1371/journal.pone.0356302.r006

**Authors:** Yongzhong Guo

PONE-D-25-69189R2

PLOS One

Dear Dr. Marrufo,

I'm pleased to inform you that your manuscript has been deemed suitable for publication in PLOS One. Congratulations! Your manuscript is now being handed over to our production team.

At this stage, our production department will prepare your paper for publication. This includes ensuring the following:

* All references, tables, and figures are properly cited

* All relevant supporting information is included in the manuscript submission,

* There are no issues that prevent the paper from being properly typeset

You will receive further instructions from the production team, including instructions on how to review your proof when it is ready. Please keep in mind that we are working through a large volume of accepted articles, so please give us a few days to review your paper and let you know the next and final steps.

Lastly, if your institution or institutions have a press office, please let them know about your upcoming paper now to help maximize its impact. If they'll be preparing press materials, please inform our press team within the next 48 hours. Your manuscript will remain under strict press embargo until 2 pm Eastern Time on the date of publication. For more information, please contact onepress@plos.org.

You will receive an invoice from PLOS for your publication fee after your manuscript has reached the completed accept phase. If you receive an email requesting payment before acceptance or for any other service, this may be a phishing scheme. Learn how to identify phishing emails and protect your accounts at https://explore.plos.org/phishing.

If we can help with anything else, please email us at customercare@plos.org.

Thank you for submitting your work to PLOS One and supporting open access.

Kind regards,

PLOS One Editorial Office Staff

on behalf of

Dr. Yongzhong Guo

Academic Editor

PLOS One
